# Identification and validation of the miRNA–mRNA regulatory network in fetoplacental arterial endothelial cells of gestational diabetes mellitus

**DOI:** 10.1080/21655979.2021.1950279

**Published:** 2021-07-07

**Authors:** Longkai He, Xiaotong Wang, Ya Jin, Weipeng Xu, Yi Guan, Jingchao Wu, Shasha Han, Guosheng Liu

**Affiliations:** Department of Pediatrics, The First Affiliated Hospital of Jinan University, Guangzhou, Guangdong, China

**Keywords:** Mirna-mRNA network, fetoplacental endothelial cells, gestational diabetes mellitus, heart development, cardiac hypertrophy, bioinformatics analysis

## Abstract

Gestational diabetes mellitus (GDM) increases the risk of fetal heart malformations, though little is known about the mechanism of hyperglycemia-induced heart malformations. Thus, we aimed to reveal the global landscape of miRNAs and mRNAs in GDM-exposed fetoplacental arterial endothelial cells (dAECs) and establish regulatory networks for exploring the pathophysiological mechanism of fetal heart malformations in maternal hyperglycemia. Gene Expression Omnibus (GEO) datasets were used, and identification of differentially expressed miRNAs (DEMs) and genes (DEGs) in GDM was based on a previous sequencing analysis of dAECs. A miRNA-mRNA network containing 20 DEMs and 65 DEGs was established using DEMs altered in opposite directions to DEGs. In an in vivo study, we established a streptozotocin-induced pregestational diabetes mellitus (PGDM) mouse model and found the fetal cardiac wall thickness in different regions to be dramatically increased in the PGDM grouValidation of DEMs and DEGs in the fetal heart showed significantly upregulated expression of let-7e-5p, miR-139-5p and miR-195-5p and downregulated expression of SGOL1, RRM2, RGS5, CDK1 and CENPA. In summary, we reveal the miRNA-mRNA regulatory network related to fetal cardiac development disorders in offspring, which may shed light on the potential molecular mechanisms of fetal cardiac development disorders during maternal hyperglycemia.

## graphic abstract

**Figure:**
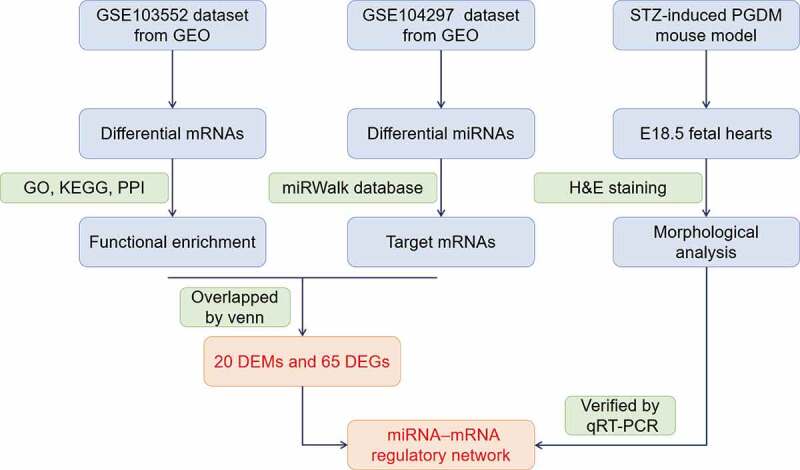

## Introduction

The risk of birth defects in the offspring of women with gestational diabetes mellitus (GDM) is significantly higher than that of normal pregnant women [1]. Children of women with diabetes have 3.4 times higher rates of congenital heart disease [2]. This may be because the development of the cardiovascular system occurs in the early stages of embryonic development, and thus, it is more susceptible to harmful factors in the uterus. These congenital heart diseases include atrial septal defects, ventricular septal defects, cardiac hypertrophy, and tetralogy of Fallot, among others [3]. However, the etiology and pathogenesis of congenital heart defects caused by GDM have not yet been fully elucidated.

The placenta is the location of fetal-maternal nutrient and metabolite exchange and is crucial for fetal development [4]. Human fetoplacental endothelial cells are an ideal model for studying epigenetic functions because of their indirect contact with fetal circulation [5]. Many articles have reported that the abnormal structure and function of fetoplacental endothelial cells leads to an increased risk of congenital heart disease in offspring [6,7]. Therefore, it is necessary to clarify the role of fetoplacental endothelial cells in fetal cardiovascular development.

MicroRNAs (miRNAs) are abundant small noncoding RNAs that regulate the expression of more than 60% of mRNAs. miRNAs silence gene expression by binding to the 3ʹ untranslated regions (3ʹUTRs) of their target mRNAs, leading to mRNA degradation or translational inhibition [8]. Recent studies have suggested that miRNAs can be released from the placenta to maternal and fetal circulation as early as the 6th week of gestation [9,10]. Studies have also found that miRNAs are involved in regulating heart development and may serve as a biomarker of abnormal heart development [11,12]. However, the regulatory mechanism of placental vascular endothelial cell miRNAs on fetal heart development in pregnant women with GDM remains to be fully elucidated.

Therefore, we hypothesize that changes in expression of the GDM transcriptome will affect the heart development of offspring. The aim of this study was to understand the potential pathogenic mechanism of GDM in abnormal heart development in offspring. We constructed and verified a novel miRNA-mRNA regulatory network that plays an important role in abnormal heart development in the offspring of mothers with GDM.

## Materials and methods

### Data acquisition

miRNA sequencing (miRNA-seq) and mRNA microarray datasets were obtained from the Gene Expression Omnibus (GEO, http://www.ncbi.nlm.nih.gov/geo/) database. The miRNA-seq dataset (GSE104297) was deposited by Strutz J et al [13] and was composed of 14 GDM-exposed fetoplacental arterial endothelial cell (dAEC) samples and 14 control samples. The mRNA microarray dataset (GSE103552) was provided by Cvitic S et al [14] and was composed of 11 dAEC samples and 8 control samples. The GSE103552 dataset was generated by GPL6244 (Affymetrix, USA).

### Data processing and identification of differentially expressed miRNAs and genes

For the miRNA-seq data from GEO, the ‘edgeR’ package in R was used to screen differentially expressed miRNAs (DEMs) as previously described [13]. For the mRNA data from GEO, the ‘limma’ package in R was used for background correction, normalization and differentially expressed gene (DEG) screening. A false discovery rate (FDR)<0.05 and [log2 (fold change)]>1 were selected as the cutoff values for identifying significant DEGs [15].

### Functional and signaling pathway enrichment analysis

Functional annotation of Gene Ontology (GO) and Kyoto Encyclopedia of Genes and Genomes (KEGG) pathway analyses of the mRNA data was performed with the ‘clusterProfiler’ package in R. An FDR<0.05 was considered statistically significant [16,17].

### Construction of the miRNA–mRNA regulatory network

The target genes of DEMs were predicted from the miRWalk database (http://mirwalk.umm.uni-heidelberg.de). The miRWalk database mainly collects information from 13 types of existing miRNA-mRNA regulatory relationship prediction algorithms (such as TargetScan, miRDB, miRTarBase and TarPmiR) to generate prediction results [18]. DEGs and DEMs that have a regulatory relationship were selected to construct the network. Cytoscape software version 3.1.0 was used to establish and visualize the miRNA‑mRNA regulatory network. The STRING database (https://string-db.org) was used for protein–protein interaction (PPI) network analysis [19]. Cytoscape software was used to establish and visualize the PPI network. PPI node pairs with a comprehensive score > 0.4 were considered significant [20]. Then, to obtain hub genes, the degree of connectivity in the PPI network was determined using Cytoscape software. Bar plots of node pairs were exported from GraphPad Prism software version 7 (La Jolla, CA, USA).

### Animals

Eight-week-old female C57BL/6 J mice (Hua Fukang Biotechnology Company, Beijing, China) were injected with 75 mg/kg STZ (Sigma-Aldrich, USA) intraperitoneally for three consecutive days to induce diabetes mellitus. STZ was dissolved in 0.01 mol/l citrate buffer at a pH of 4.5. Seven days after STZ injection, the blood glucose level was measured by the Roche Accu-Chek Aviva Blood Glucose System (Roche, USA). A fasting blood glucose level > 288 mg/dl (16 mmol/l) indicated the successful modeling of diabetes mellitus [21,22]. The mice in the control group were injected with an equal volume of citrate buffer and maintained normal fasting blood glucose levels (4–8 mmol/l) before and during pregnancy. Pregnancy was designated E0.5 when vaginal plugs were observed after mating. During pregnancy, the mice were monitored for random blood glucose levels every 6 days. At E18.5, after the pregnant mice were sacrificed by cervical dislocation, the embryos were removed by cesarean section.

### Morphological analysis

To examine whether maternal hyperglycemia altered the morphology of the heart, the embryo hearts were fixed in 4% paraformaldehyde in PBS. The hearts were then dehydrated, embedded in paraffin wax, and serially sectioned at a thickness of 5 μm for further hematoxylin and eosin (H&E) staining. The slices were imaged using a microscope (Olympus BX53, Tokyo, Japan) equipped with cellSens Standard 1.9 software. The average thicknesses of the right ventricular wall (RVW), ventricular septum (VS), left ventricular wall (LVW) and trabeculae were measured as previously described [23]. A minimum of six random images from six samples were assayed per group.

### RNA isolation and quantitative PCR

Total RNA was extracted from mouse embryo hearts using RNAiso Plus reagent (TaKaRa, Japan). cDNA was synthesized using a RevertAid First Strand cDNA Synthesis Kit (Thermo Scientific, USA). Gene expression was quantified using TB Green Premix Ex Taq II (TaKaRa, Japan), a CFX96 Touch Real-Time PCR Detection System (Bio-Rad, USA) and CFX Manager Software (Bio-Rad, USA). The cDNAs were amplified at 95°C for 2 min for the initial denaturation step, followed by 40 PCR cycles (95°C for 15 seconds, 60°C for 30 seconds). β-Actin was used as the internal reference of DEGs, and U6 was regarded as the internal reference of DEMs. The primers are listed in Table S1.

### Data analysis

The SPSS 20.0 statistical software package program (IBM, Armonk, NY, USA) was used for statistical analysis. GraphPad Prism 7 software (La Jolla, CA, USA) was used to construct statistical graphs. Data are presented as the means ± SD. The Student’s t-test or Mann-Whitney U-test was conducted for analysis of the two groups. Significance was assumed when *p* < 0.05.

## Results

### Identification of DEMs and DEGs

GDM increases the risk of fetal heart malformations. Mounting evidence shows that miRNAs from maternal blood vessels can be passed to the fetus through the placenta, thereby affecting the development of various embryonic systems. However, little is known about the role of the miRNA-mRNA regulatory network in embryonic heart malformations caused by GDM. The purpose of this study was to build a miRNA-mRNA regulatory network to help improve the current understanding of the pathophysiological mechanisms of the miRNA-mRNA axis in GDM, especially in cardiovascular development.

**Figure 1.**
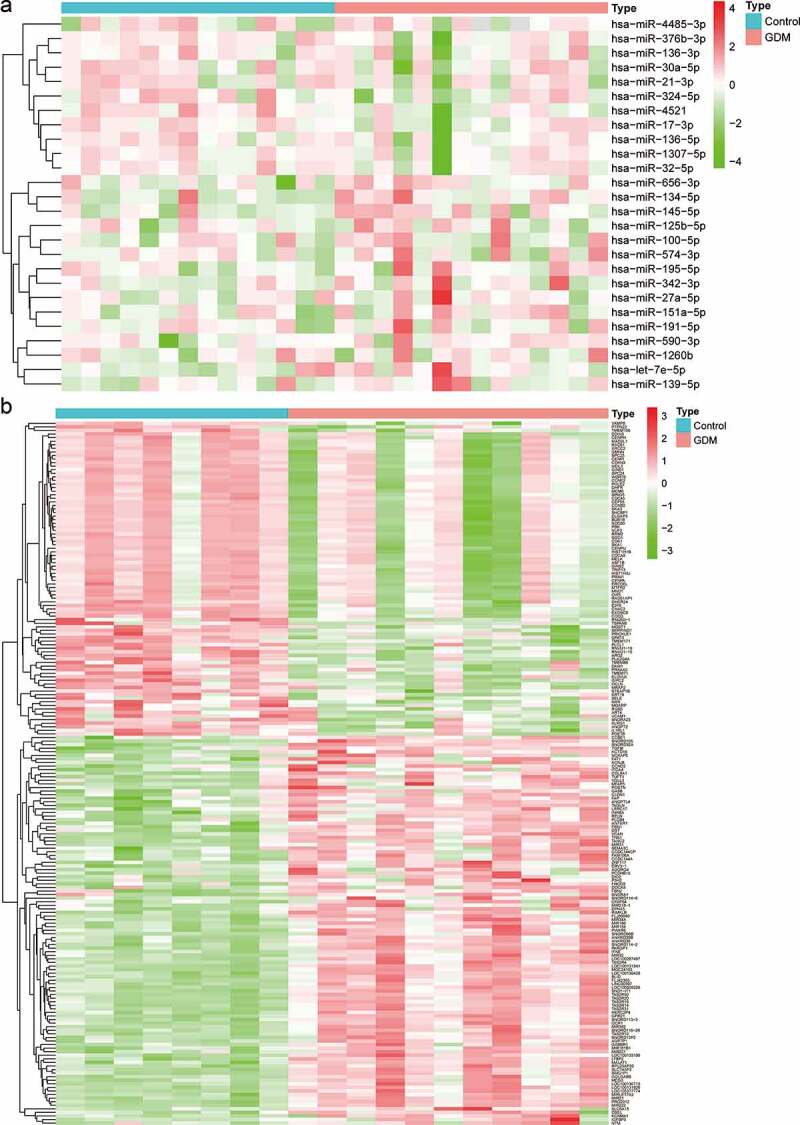
Identification of differentially expressed miRNAs (DEMs) and mRNAs (DEGs). (a) Heatmap of DEMs among the control and GDM groups. (b) Heatmap of DEGs among the control and GDM groups

The miRNA-seq profiles (GSE104297) consisted of a total of 11 dAEC samples and 8 normal samples. A total of 26 DEMs were identified, of which 11 were downregulated and 15 were upregulated (Figure 1a and Table S2). The gene expression profiles (GSE103552) consisted of a total of 14 dAEC samples and 6 normal samples. According to the criteria of FDR<0.05 and [log2 (fold change)]>1, a total of 109 upregulated and 88 downregulated DEGs were obtained (Figure 1b and Table S3).

### Functional enrichment analysis

**Figure 2.**
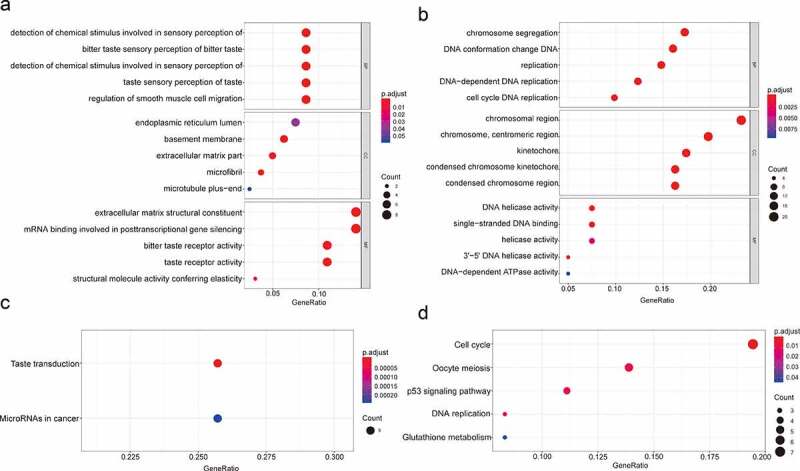
GO functional annotation and KEGG pathway analysis. (a) The top 5 enriched GO functional annotations of upregulated DEGs. (b) The top 5 enriched GO functional annotations of downregulated DEGs. (c) The top 5 enriched KEGG pathways of upregulated DEGs. (d) The top 5 enriched KEGG pathways of downregulated DEGs

GO enrichment analysis suggested that the biological processes of the upregulated genes are mainly related to ‘sensory perception of bitter taste’ and ‘positive regulation of smooth muscle cell migration’. The cellular components of the upregulated genes are mainly associated with ‘endoplasmic reticulum lumen’, ‘basement membrane’ and ‘extracellular matrix part’. Regarding molecular functions, the upregulated genes were mostly enriched in the terms ‘extracellular matrix structural constituent’, ‘mRNA binding involved in posttranscriptional gene silencing’ and ‘bitter taste receptor activity’ (Figure 2a). The biological processes of the downregulated genes mainly showed enrichment for ‘chromosome segregation’, ‘DNA conformation change’ and ‘DNA replication’. For cellular components, the downregulated genes are primarily related to ‘chromosomal region’, ‘centromeric region’ and ‘kinetochore’, and ‘DNA helicase activity’, ‘single-stranded DNA binding’ and ‘helicase activity’ were associated with downregulated genes molecular functions (Figure 2b).

The enriched KEGG pathways for upregulated genes included ‘Taste transduction’ and ‘MicroRNAs in cancer’ (Figure 2c). The enriched KEGG pathways for downregulated genes included ‘Cell cycle’, ‘Oocyte meiosis’, ‘p53 signaling pathway’, ‘DNA replication’ and ‘Glutathione metabolism pathway’ (Figure 2d).

### Construction of the miRNA‑mRNA regulatory network

**Figure 3.**
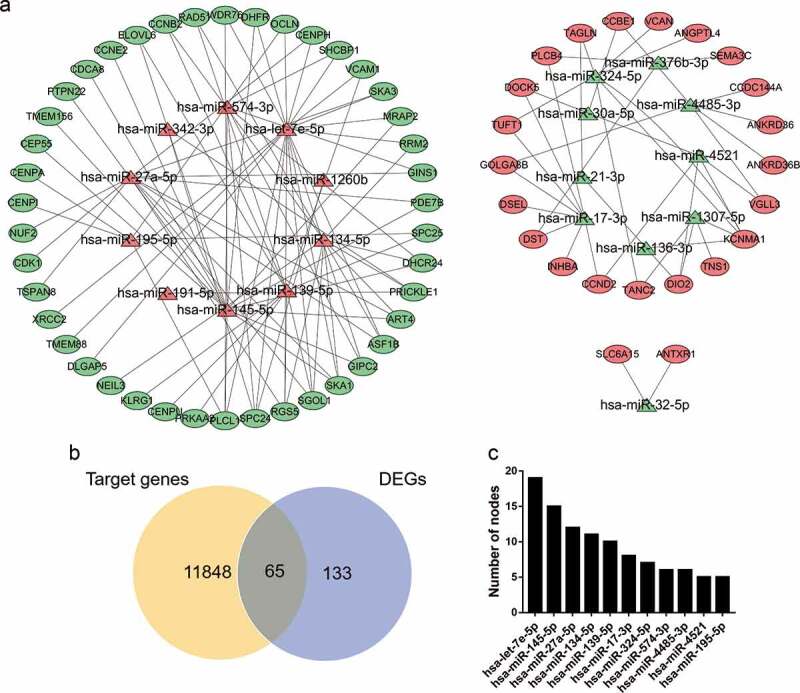
miRNA-mRNA regulatory network. (a) Red triangles represent upregulated miRNAs, green triangles represent downregulated miRNAs, red ovals represent upregulated mRNAs, green ovals represent downregulated mRNAs and lines represent the interactions between DEMs and DEGs. (b) Venn diagram showing overlapping genes between target genes and DEGs. (c) Top 10 hub miRNAs with the highest degree of connectivity

A total of 11,913 miRNA‑mRNA regulatory pairs were identified via the miRWalk platform. The miRNA‑mRNA regulatory pairs included 65 genes overlapping with the DEGs (Figure 3b). These overlapping genes were regulated by 20 DEMs. Afterward, a regulatory network between the 20 DEMs and 65 DEGs was constructed and visualized (Figure 3a). The specific miRNA-mRNA interaction sequence is listed in Table S4. The top ten hub miRNAs listed in Figure 3c were identified according to the node degree. Among them, the upregulated miRNAs were hsa-let-7e-5p, hsa-miR-145-5p, hsa-miR-27a-5p, hsa-miR-134-5p, hsa-miR-139-5p, hsa-miR-574-3p and hsa-miR-195-5p, and the downregulated miRNAs were hsa-miR-17-3p, hsa-miR-324-5p, hsa-miR-4485-3p and hsa-miR-4521.

### PPI network analysis

**Figure 4.**
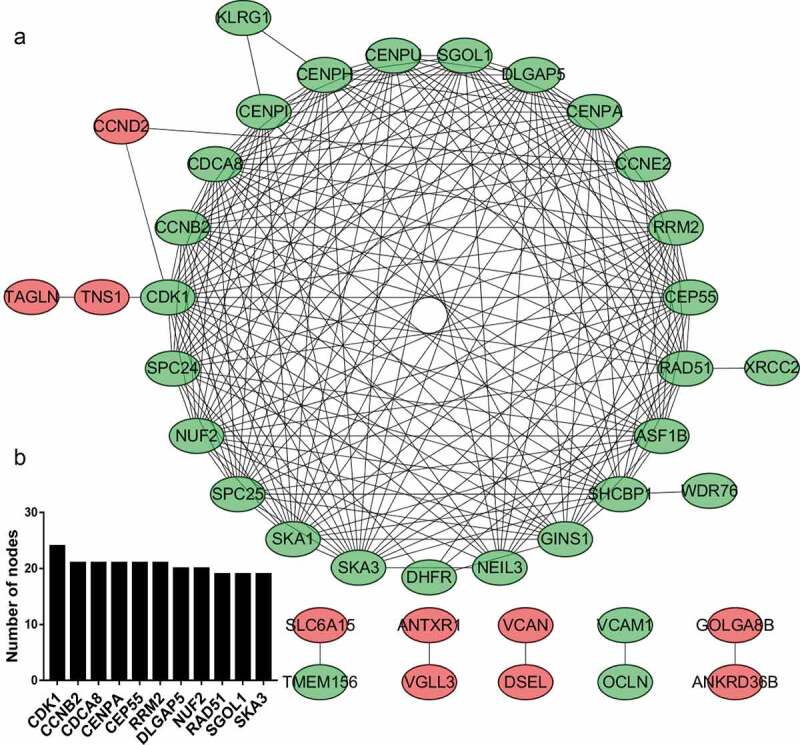
PPI network and module analysis. (a) PPI network of DEGs. The red ovals represent upregulated genes, and the green ovals represent downregulated genes. (b) Top 10 hub genes with the highest degree of connectivity

Based on the enrichment results of the STRING database, many downregulated target genes were found to interact with each other (Figure 4a). To better visualize the results, the top 10 hub nodes with the highest degrees were screened, all of which were downregulated target genes (Figure 4b).

### Cardiac hypertrophy in embryonic mice with mothers having pregestational diabetes mellitus (PGDM)

To investigate whether GDM has a negative impact on the embryonic cardiovascular system, we established a PGDM mouse model by the intraperitoneal injection of STZ [22]. The fasting blood glucose of the mice was monitored to determine whether the diabetic mouse model was successfully established.

**Figure 5.**
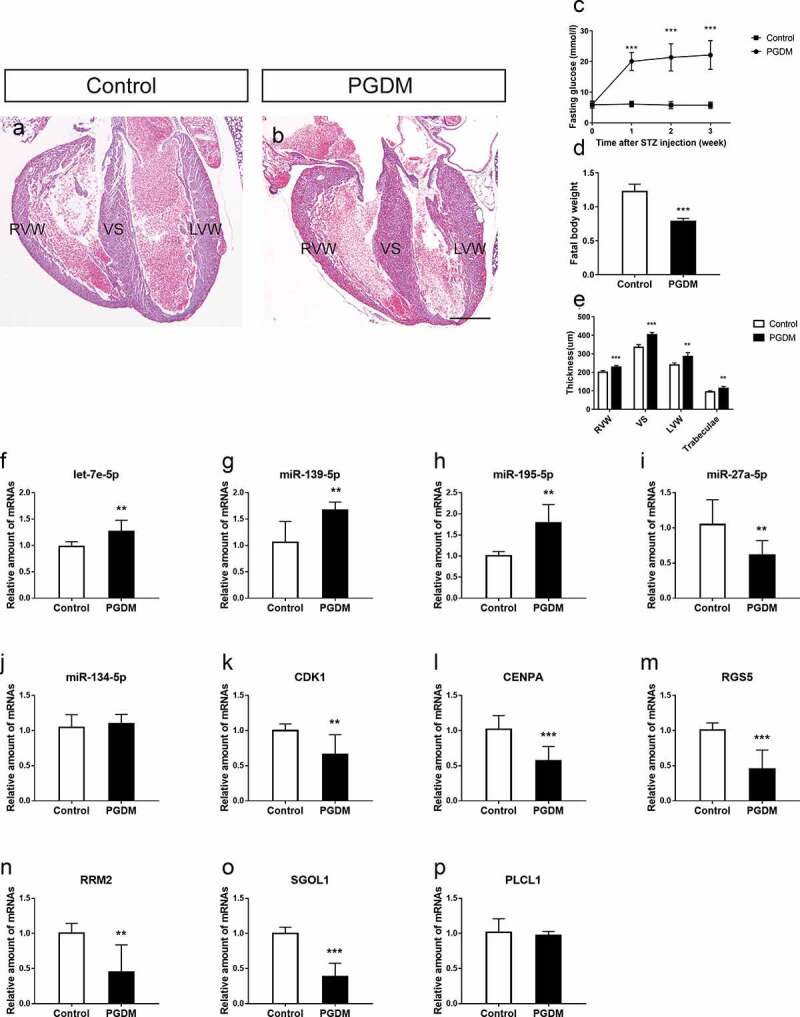
Validation of hub miRNAs and genes in the fetal heart of the control and PGDM groups. (a-b) H&E staining of E18.5 mouse heart vertical sections in the control (a) and PGDM (b) groups. (c) The detection of mouse maternal glucose blood in weeks 0–3. (d) The weight of fetuses in the control and PGDM groups at E18.5. (e) Quantification of the thicknesses of the RVW, VS, LVW and trabeculae of mouse hearts in the control and PGDM groups at E18.5. (f-p) Validation of hub miRNAs and genes in the fetal hearts of the control and PGDM groups using quantitative real-time PCR. Scale bars = 400 μm. *p < 0.05, **p < 0.01, ***p < 0.001

One week after three consecutive intraperitoneal injections of STZ, the blood glucose level of the diabetes mellitus group increased from 5.99 ± 1.15 mmol/l (n = 10) to 20.05 ± 2.96 mmol/l (n = 10), which was significantly higher than that of the control group (6.13 ± 0.93 mmol/l, n = 10; *p* < 0.001, Figure 5c). This result indicates that we successfully established a mouse model of maternal diabetes. The blood glucose level of diabetic mice remained at a high level (over 16.0 mmol/l) throughout pregnancy.

Importantly, the blood glucose level in the control group did not increase (Figure 5c). The body weight of PGDM fetal mice on day E18.5 was significantly lower than that of control mice (Figure 5d). To determine whether the offspring of PGDM mice had cardiac malformations, we used H&E staining to monitor the heart development of E18.5 fetal mice. We found that in the same developmental stage, PGDM mice had hypertrophic hearts, and the cardiac cavity decreased in comparison with that in the control mice (Figure 5a-b). By measuring the different regions of the cardiac wall, we found that the RVW of the PGDM group (228.48 ± 8.42.19 μm, n = 6) was significantly thicker than that of the control group (200.95 ± 8.53 μm, n = 6, *p* < 0.001), the VS of the PGDM group (403.66 ± 12.53 μm, n = 6) was significantly thicker than that of the control group (335.77 ± 14.55 μm, n = 6, *p* < 0.001), and the LVW of the PGDM group (286.02 ± 20.91 μm, n = 6) was significantly thicker than that of the control group (239.66 ± 11.24 μm, n = 6, *p* < 0.01). Finally, the trabeculae of the PGDM group (113.19 ± 10.16 μm, n = 6) were significantly thicker than those of the control group (94.34 ± 5.84 μm, n = 6, *p* < 0.01, Figure 5e). These results suggest that maternal hyperglycemia caused cardiac hypertrophy of the developing fetus.

### qRT-PCR validation of DEGs and DEMs in fetal mouse hearts

According to the results of PPI network analysis, stronger interactions were observed in downregulated mRNAs than in upregulated mRNAs. Therefore, we only verified the expression of specific upregulated miRNAs and downregulated mRNAs selected based on the node degree. To validate the microarray results, the expression levels of DEMs (let-7e-5p, miR-139-5p, miR-27a-5p, miR-134-5p and miR-195-5p) and corresponding hub target genes (CDK1, CENPA, PLCL1, RGS5, RRM2 and SGOL1) were quantified in the E18.5 fetal hearts of the PGDM group and the control grouLet-7e-5p expression was significantly upregulated in the PGDM group (*p* < 0.01), and its target genes SGOL1, RRM2 and RGS5 tended to be downregulated in the PGDM group (figure 5f, M-O). miR-139-5p showed an upregulated expression trend, and its corresponding target gene SGOL1 tended to be downregulated in the PGDM group (Figure 5g, o). miR-195-5p showed an upregulated expression trend, and its corresponding target gene CENPA tended to be downregulated in the PGDM group (Figure 5h, l). The expression levels of miR-27a-5p, miR-134-5p and PLCL1 were not consistent with the bioinformatics analysis (Figure 5i, J, P).

## Discussion

A previous meta-analysis showed that GDM can cause fetal cardiac hypertrophy, diastolic dysfunction, and myocardial damage [24], yet the specific cellular and molecular mechanisms are poorly understood. Disorders of the miRNA-mRNA regulatory network in the placenta can cause many congenital abnormalities, such as neural tube defects [25], ventricular septal defects [12], smaller head circumference [26] and acute fetal hypoxia [27]. Previous research has only focused on the effects of miRNAs or mRNAs in GDM on the heart development of offspring, without a comprehensive analysis of the miRNA-mRNA axis [28–30]. Strutz J [13] and Cvitic S [14] analyzed the changes in the expression of miRNAs and mRNAs in dAECs of GDM and concluded that these changes would increase the risk of long-term cardiovascular disease in the offspring. However, the study did not further clarify and verify whether these miRNAs and mRNAs were involved in the development of the heart during the embryonic stage. Furthermore, to the best of our knowledge, a comprehensive miRNA-mRNA regulatory network for hyperglycemia during pregnancy has not been established thus far. Therefore, it is essential to construct a miRNA-mRNA regulatory network to further study the molecular mechanism by which maternal hyperglycemia causes fetal cardiac hypertrophy, which is the key aim of this research.

In this study, we analyzed the differential expression levels of miRNAs and mRNAs in dAEC samples using the GEO datasets GSE103552 and GSE104297, and 20 DEMs and 65 key DEGs were ultimately obtained. GO and KEGG enrichment analyses demonstrated that DEGs are involved in the cell cycle and DNA replication. Recent studies have indicated that low expression of cell cycle driver factors inhibits the cell cycle of cardiomyocytes [31]. The combined low expression of CDK1/cyclin B1 and CDK4/cyclin D1 inhibited the cycle of cardiomyocytes after mitosis [32]. Myocardial TBX20 directly regulates related genes required for DNA replication in fetal cardiomyocytes. In Tbx20-knockout mice, the circumference of developing atrial and ventricular chambers was significantly reduced [33]. Therefore, correct expression of cell cycle- and DNA replication-related factors plays an important role in the proliferation and development of cardiomyocytes.

Based on the results of bioinformatics analysis, we constructed a miRNA‐mRNA regulatory network to help us understand the possible molecular mechanisms of cardiac malformation in the offspring of mothers with GDM. In the miRNA-mRNA regulatory network, the upregulated DEMs had the largest number of target DEGs. According to the PPI network, the top ten hub genes were all downregulated. According to previous studies, most of the DEGs and DEMs that we identified are involved in cardiovascular development. For example, hsa-let-7e-5p is overexpressed in pathological cardiac hypertrophy [34], heart failure [35] and congenital heart disease [36]. hsa-let-7e-5p had the most predicted target genes, of which SGOL1, RRM2, RGS5 and PLCL1 have been shown to correlate with cardiovascular development. SGOL1 encodes a component of the cohesin complex, and its missense mutation has been found to be associated with the occurrence of chronic atrial arrhythmia through accelerated cell cycle progression and enhanced activation of TGF-β signaling [37]. RRM2 encodes one of two nonidentical subunits of ribonucleotide reductase, and its low expression inhibits myocardial contractility [38]. RGS5 encodes a member of the regulators of the G protein signaling (RGS) family and plays an important role in cardiovascular development [39]. RGS5 prevents cardiac hypertrophy through inhibition of MEK-ERK1/2 signaling, especially in hyperglycemic environments [40,41]. Genome-wide association studies have indicated that PLCL is associated with cardiovascular disease risk [42,43]. Upregulation of miR-139-5p expression is associated with atrial septal defects by targeting a mutation (c. *1784 T > C) in the 3ʹUTR of ACTC1 [44]. The predicted target genes of miR-139-5p are SGOL1, RAD51 and CCNB2, though there is no research showing that RAD51 and CCNB2 have a direct relationship with heart development. High expression of miR-195-5p might promote hypertrophy by targeting MFN2 and FBXW7 [45]. The predicted target genes of miR-139-5p are SPC25, CENPI and CENPA. CENPA encodes a centromere protein and is necessary for maintaining proliferation, inhibiting senescence, and promoting survival following differentiation of cardiac progenitor cells [46]. Cardiac fibroblast miR-27a-5p may function as an inhibitor of pathological myocardial fibrosis and hypertrophy by negatively regulating Egr3 expression [47]. The predicted target genes of miR-27a-5p are SGOL1, NUF2, CDK1 and ASF1B. Low expression of CDK1 is associated with the occurrence of myocardial defects and ventricular hypoplasia by reducing the expression of the Cdk1 activator Cdc25B phosphatase and the NFATc3 TF [48]. High expression of miR-134-5p promotes myocardial apoptosis and angiogenesis after myocardial infarction by targeting XIAP and KDM2A [49,50]. The predicted target genes of miR-134-5p are SGOL1, RRM2, RAD51 and CCNB2, but other hub DEMs and DEGs in cardiovascular development have not been studied.

In addition, we successfully established an STZ-induced PGDM model. Further research revealed that the RVW, VS, LVW and trabecular thicknesses of the hearts of E18.5 mice increased significantly when hyperglycemia occurred during pregnancy. We validated the expression of these DEMs and their target genes in fetal heart tissues. QPCR data revealed that the expression of let-7e-5p, miR-139-5p and miR-195-5p was significantly upregulated in the PGDM group, and the expression of their target genes SGOL1, RRM2, RGS5, CDK1 and CENPA decreased significantly, which is consistent with the results of our bioinformatics analysis. Therefore, these DEMs and their target genes may be involved in the pathogenic mechanisms leading to cardiovascular development malformations in the offspring of GDM mothers. Nevertheless, the targeting relationship of these identified miRNA-mRNA interactions requires further validation to understand the effect of placental vascular endothelial cells on fetal cardiovascular development in maternal hyperglycemia. This study has several limitations. First, the number of fetoplacental AEC samples was small, which may affect the reliability of our results. Second, we used P values instead of FDR to screen DEMs; when we used FDR as the cutoff, only a few miRNAs were identified. Therefore, the P value used in this study may be biased. Third, we did not conduct specific cellular and molecular biological experiments of this miRNA-mRNA regulatory network under physiological and pathological conditions of cardiac development.

## Conclusions

First, we systematically analyzed the expression levels of miRNAs and mRNAs in dAECs and constructed a corresponding miRNA-mRNA regulatory network to study their potential role in maternal hyperglycemia during heart development in offspring. To verify the miRNA-mRNA regulatory network, we constructed a PGDM mouse model and found that PGDM caused cardiac hypertrophy in mouse fetal hearts. Finally, we identified some candidate miRNAs and mRNAs in the miRNA-mRNA regulatory network that are closely related to cardiovascular development. Overall, we produced a miRNA–mRNA regulatory network that provides novel ideas and methods for achieving a deeper understanding of fetal cardiac hypertrophy during maternal hyperglycemia and also establishes a foundation for further research on normal heart development.

## Supplementary Material

Supplemental Material

## Data Availability

All analyzed data are from the GEO database. The original data are available upon request to the corresponding author.
